# Difficulties when Assessing Birdsong Learning Programmes under Field Conditions: A Re-Evaluation of Song Repertoire Flexibility in the Great Tit

**DOI:** 10.1371/journal.pone.0016003

**Published:** 2011-01-17

**Authors:** Hector F. Rivera-Gutierrez, Rianne Pinxten, Marcel Eens

**Affiliations:** Ethology Group, University of Antwerp, Wilrijk, Belgium; CNRS - University Paul Sabatier, Toulouse, France

## Abstract

There is a remarkable diversity of song-learning strategies in songbirds. Establishing whether a species is closed- or open-ended is important to be able to interpret functional and evolutionary consequences of variation in repertoire size. Most of our knowledge regarding the timing of vocal learning is based on laboratory studies, despite the fact that these may not always replicate the complex ecological and social interactions experienced by birds in the wild. Given that field studies cannot provide the experimental control of laboratory studies, it may not be surprising that species such as the great tit that were initially assumed to be closed-ended learners have later been suggested to be open-ended learners. By using an established colour-ringed population, by following a standardized recording protocol, and by taking into account the species' song ecology (using only recordings obtained during peak of singing at dawn), we replicated two previous studies to assess song repertoire learning and flexibility in adult wild great tits elicited by social interactions. First, we performed a playback experiment to test repertoire plasticity elicited by novel versus own songs. Additionally, in a longitudinal study, we followed 30 males in two consecutive years and analysed whether new neighbours influenced any change in the repertoire. Contrary to the previous studies, song repertoire size and composition were found to be highly repeatable both between years and after confrontation with a novel song. Our results suggest that great tits are closed-ended learners and that their song repertoire probably does not change during adulthood. Methodological differences that may have led to an underestimation of the repertoires or population differences may explain the discrepancy in results with previous studies. We argue that a rigorous and standardized assessment of the repertoire is essential when studying age- or playback-induced changes in repertoire size and composition under field conditions.

## Introduction

In most songbird species, males exhibit a song repertoire of variable size and composition, which has been shown to play a crucial role in female attraction and/or territorial defence [1], [2], [3]. Interspecific differences in repertoire size are mainly mediated by a large diversity of song-learning programmes that determine when the songs are learnt and for how long the song repertoire is modified [4], [5]. The duration of the sensitive period for memorizing songs varies from a month (or less) during the first months of life (e.g. Zebra finch *Taeniopygia guttata* [6]) to throughout life (e.g. European starling *Sturnus vulgaris* [7], [8], [9]). Bird species that learn the songs during a limited short period early in life and do not change their crystallized repertoire as adults are known as closed-ended (or age-limited) learners [4], [5], [10], [11]. On the other hand, species that are able to add new songs during adulthood are classified as open-ended learners [4], [5], [10]. Establishing whether a species is closed- or open-ended is important to enable interpretation of functional or evolutionary (fitness) consequences of variation in repertoire size. For instance, song learning in open-ended learners may allow song repertoire to serve as a more immediate indicator of a male's condition, and not just as an indicator of his condition as nestling or young fledgling [12].

In order to establish unambiguously whether a songbird can memorize new songs during adulthood, controlled laboratory experiments are necessary. However, social and ecological factors that are not present during laboratory experiments may provide key variables for the understanding of birdsong learning [13]. Therefore, field studies could offer an alternative approach to enable us to establish when birds memorize their repertoires [13], [14]. One of the main difficulties in identifying a species as an age-limited or open-ended learner under field conditions is the labour-intensive method of documenting full song repertoires in multiple years or after experimental treatment [14]. In addition, since field studies cannot provide the experimental control of laboratory studies, it is important to use individually marked birds under a common and standardized recording protocol.

The difficulty in establishing the timing of song repertoire learning in wild populations can be well illustrated using the great tit (*Parus major*). Although this species was initially classified as a closed-ended learner [15], some evidence suggests, however, that great tit males may be able to modify their repertoire as adults [16], [17], mainly mediated by social interactions with new neighbours in successive years [17]. In addition, a recent playback study surprisingly suggested that repertoire size and composition were very flexible within a season in adult great tits after exposure to novel songs [18]. These findings seem to indicate that contrary to the previous suggestions, the species is an open-ended learner. Although field studies have provided a major impetus to the study of social factors in song learning, there is still little understanding in the field of exactly how social variables shape song learning [11].

Given that repertoire size and composition have often been considered as a measurement of individual male quality in songbirds [19], [20], Franco & Slabbekoorn's surprising findings on repertoire plasticity may have major implications for sexual selection studies, and based on their results they argued for the conceptual reconsideration of the role of repertoire size as a signal of male quality [18]. In this study, we developed a standardized recording protocol to replicate the two previous field studies that provided evidence for song repertoire flexibility and open-ended learning in the great tit elicited by social interactions [17], [18]. First, we examined within season song repertoire flexibility in adult great tits and in line with Franco & Slabbekoorn [18] we developed a playback experiment to test repertoire plasticity elicited by novel versus own songs. In contrast to their study, we used a colour-ringed nest box breeding population, enabling not only individual recognition at any time during the experiment but also a much more standardized song recording protocol (see below). We predicted that if great tit males express any degree of song repertoire plasticity, individuals receiving unfamiliar song will try to match the ‘new’ song by including new songs in their repertoire or recalling unsung songs from memory [18]. On the contrary, birds receiving their own song will match the playback [21], [22] and no ‘new’ songs will appear. Significant differences in repertoire size and composition will, therefore, appear both between groups (own – unfamiliar song) and between the first and the second dawn chorus. Our second objective was to establish whether adult great tit males are able to add new songs after the first year of life. Using McGregor & Krebs' study [17] as an example, we performed a longitudinal analysis following colour-ringed males for two years to determine whether repertoire size and/or composition change between years in adult males. We predicted that individuals will learn new songs from new neighbours, and as a result, repertoire size and composition will differ significantly between the first and second year. An important final difference between our study and the two previous studies [17], [18] is that, in both the experimental and longitudinal study, we always recorded the entire dawn chorus, which has previously been shown to provide a repeatable estimation of the song repertoire [23].

## Materials and Methods

### Ethics Statement

The study was performed under proper legislation of the Belgian and Flemish law and was approved by the ethical committee of the University of Antwerp (ID number 2006/22). The Belgian Royal Institute for Natural Sciences (Koninklijk Belgisch Instituut voor Natuurwetenschappen) provided ringing licences for the authors and technical personnel. The methods that we used (song recording, nest checking, ringing, playback experiment) created no (or only a very low level of) stress and did not cause any desertion from nestling activity or mortality.

### Study area and general procedures

This study was carried out in a suburban great tit population located on campus Drie Eiken of the University of Antwerp, Belgium, which has been studied since 1997 [23]. Great tits are cavity-breeders that readily accept nest boxes to breed in spring and to sleep during winter. The population was monitored all year round and breeding stages (nest building, egg-laying, incubation, feeding) were established for all couples breeding in the nest boxes. All individuals received a metallic-numbered ring when first caught while sleeping in nest boxes or when handled as nestlings. Adult birds were also given a combination of three colour rings to allow individual identification in the field. Hence, the identity of all individuals was known before the recordings and the playback experiment were carried out. As age was included to control the statistical analysis, we determined exact age using birth records for all resident birds. Age of immigrant birds was determined based on colour differences of primary coverts, grey for yearlings and bluish for older birds [24]. Exact age could not be determined for four individuals that arrived as adults, so their age was not included in the analysis.

### Song recordings and analysis

Great tit males sing year-round, but singing activity increases during the breeding season [25] and it is highly related to female fertility [26]. Males display a peak of uninterrupted singing activity in the surroundings of the nest before sunrise during the reproductive period, known as the dawn chorus [26], [27], which is thought to have a function for territorial defence, mate guarding, and female attraction [26], [28], [29]. Both for the playback experiment and the longitudinal analysis, we recorded the complete dawn chorus of great tit males during the egg-laying period of their females and at the earliest when the second egg was laid [23], [30]. Dawn choruses were recorded between 5:00 and 7:20 am and the person responsible for the recording was always present before the male started singing up until the dawn chorus ended. Following previous studies, we considered the end of the dawn chorus to be when the female emerged from the nest and engaged in copulation behaviour with the male [23], [30], [31].

Recordings were made during the spring of 2008 and 2009 either by using a Sennheiser Me67/K6 directional microphone attached to a portable Marantz PMD660 solid-state digital recorder or by placing an M-Audio MicroTrack 24/96 Professional Mobile Digital Recorder on the top of the nest box [30]. The recording settings were the same for both recorders: PCM, 44100 Hz, 16 Bit, Mono. All analysed recordings contained an entire dawn chorus with a clearly definable beginning and end. Duration of dawn chorus did not correlate with repertoire size for the playback experiment (*r* = 0.06, *P* = 0.8, *N* = 18) nor for the longitudinal analysis (*r* = 0.18, *P* = 0.32, *N* = 30). Recordings were submitted to spectrographic analysis using Avisoft-SASLab Pro 4.51 (Avisoft Bioacoustics, Berlin, Germany; sonogram parameters: FFT length 256, Frame size 75% and overlap of 50%) and all analyses were performed blind with respect to the identity of the individuals.

**Figure 1:**
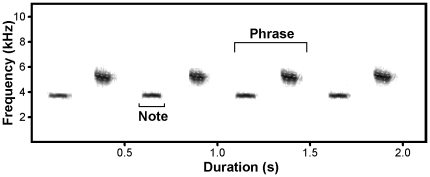
Spectrographic representation of great tit song. This figure shows a complete strophe of a two notes song type, indicating what constitutes a phrase and a note.

Great tit males have a repertoire ranging from 1 to up to 9 different discrete units or phrase types, also referred to as song types that can be recognized unambiguously [15], [32]. Song types are repeated in a stereotypic way during 1 to 5 seconds which is referred as a strophe [15], [32], and usually several strophes of the same song type are sung before another song type is introduced [32], [33] (Figure 1). For all individuals, we identified all the different song types that were sung during each dawn chorus (playback experiment/longitudinal study) and constructed a library to establish repertoire size and to compare repertoire composition. Two different observers independently inspected a subsample of the dawn choruses of the same males from 2008 with the aim of estimating repeatability of the assessment of repertoire size using analysis of variance [34]. Repeatability was 0.97 (N = 28, P<0.001), indicating that classification of song types was not observer biased.

### Playback experiment

The playback experiment was conducted between March 26 and April 16 in 2009. In total 18 males were randomly assigned to a control or treatment group. Birds were tested in sets of two (control/treatment) to correct for seasonal variation. Control birds received a playback stimulus that was made up of their own song from the first recorded dawn chorus in spring 2009, and treatment birds received the song of an unfamiliar male belonging to a population located more than 10 kilometres away from our population. Recordings of unfamiliar song stimuli were collected during the dawn chorus in spring 2007. In total we used a set of 9 different song types that did not resemble any of the songs from the repertoire of the subject males, and that were randomly assigned to the treatment subjects.

Similar to Franco & Slabbekoorn's [18] study, our playback experiment was performed in three phases, but we used a more standardized recording protocol (see above for details). Our experimental set-up was as follows: I) a complete dawn chorus was recorded for each great tit male subject before the playback (hereafter first dawn chorus); II) a 12 minutes playback consisting of 3 loops of 2 minutes song followed by 3 minutes silence was carried out during two consecutive days in the morning between 7:30 and 11:00, commencing the same day or the first day after the first dawn chorus was recorded (playback); and III) a second complete dawn chorus was recorded the following day after the second playback (second dawn chorus). Additionally, during the playback each subject was recorded with a directional microphone (Sennheiser Me67/K6) and a portable Marantz PMD660 solid-state digital recorder to identify the songs sung in response to the stimulus. We required 3 to 4 days to complete the experiment for each bird (from the first to the second dawn chorus). Several non-neighbouring birds were tested/recorded the same day.

### Playback stimuli

Stimuli (control and treatment) were created with Avisoft-SASLab Pro Software, version 4.51. Each playback stimulus was created using a single song type, which has been shown to elicit singing behaviour, song type matching and switching and production of novel songs in great tits and other species [18], [35], [36]. Each subject male received the same song type during the two trials. Complete strophes of approximately 3 seconds were selected based on the quality of the recordings (signal to noise ratio). The strophes were first trimmed, then filtered with a high-pass filter at 1500 Hz and finally normalized to an amplitude of 75% of a volt. Additionally, we inserted a silent gap of 3 s and we copied both the gap and the strophe to append them several times in order to create a 2 minutes loop song. Each loop of song was preceded by a silent gap of 3 minutes and then repeated for two more times to complete a song stimulus of 12 minutes (3 loops of 2 minutes song - 3 minutes silence). The songs were broadcasted with an Anchor MiniVox loudspeaker at an approximated distance of five meters in front of the nest box. The speaker was placed on the ground and it was connected by a 20 meters cable to an M-Audio MicroTrack 24/96 Professional Mobile Digital Recorder. The two different stimuli (own versus novel song) elicited the same behavioural response (approach distance, strophes sung in response to playback: t-test all *P*>0.1), suggesting that the stimuli only differed in type and they equally stimulated singing activity.

### Longitudinal analysis

We compared the repertoire size and composition of 30 different individuals recorded in two consecutive years (breeding seasons of 2008 and 2009) during dawn chorus (see above for details). Colour-band combinations for identification, daily observations and nest box checking during winter and spring enabled us to individually identify males, establish breeding territories and reproductive stage. Nine individuals included in the longitudinal analysis were also tested during the playback experiment (4 control/5 treatment). From these individuals only the first dawn chorus recorded in 2009 was used in the longitudinal study to avoid the effect of the playback as a confounding factor.

### Data analysis

Data were checked for normality with Kolmogorov-Smirnov test and for homogeneity of variance with Levene's test. Parametric tests were done when possible; otherwise we first tried square root transformations or applied non-parametric tests. We tested whether the number of different song types sung in response to the playback differed between groups (own/unfamiliar song) by using unpaired *t*-test. Comparisons of repertoire size between the first and second dawn chorus recorded in the framework of the playback experiment and between years were done using Linear Mixed Models (LMM) that correct for non-independence of the data. To assess the effects of the playback experiment we fitted a LMM with individuals as subject and phase (first-second dawn chorus) as repeat. In the same model, treatment and phase were included as fixed factors, and we assessed main effects and the interaction between them. Additionally, exact age (1–5) was included as a covariate and we tested the covariance of it as a random factor. In the longitudinal study we fitted a LMM including individuals as subject and year (2008–2009) as repeat. Year was included as a fixed factor, and age was included as covariate. We tested the covariance of age as a random factor.

Additionally, similar to Franco & Slabbekoorn [18], we analysed whether the total repertoire size increased by comparing the first versus the accumulated repertoire, which represented the repertoire size of the first dawn chorus recorded plus the new songs observed during the second dawn chorus recorded. This analysis was done both for the playback experiment (repertoire size first dawn chorus + new song types recorded during playback and/or second dawn chorus) and the longitudinal study (repertoire size 2008 + new song types recorded 2009) using a Wilcoxon Signed Rank test. We also analysed repeatability of repertoire with an analysis of variance [34] and we compared repeatability of repertoire composition within individuals using the Dice index of similarity [37], which makes a pairwise comparison of shared elements. Although this index was developed for population ecological studies, it has been applied before in birdsong repertoire assessments [38]. The index ranges from zero, when there is no similarity between the analysed repertoires, to one, when the repertoires are identical.

We also tested whether the likelihood of changing the repertoire between years was related to the presence of new neighbouring males. Using the information on nest box occupancy of breeding pairs from the population in spring 2008 and 2009, we elaborated a 2×2 table with the number of individuals that did/did not change (add or drop) song types, and whether the individuals did/did not have new direct neighbours in an adjacent territory. We used a *X* ^2^ test to evaluate whether changes in repertoire were related to the presence of new neighbours.

Finally, we also investigated whether there was a relationship between changes in repertoire and the initial repertoire size. For this, we divided the individuals in two groups (Change/Non-change), and we performed an unpaired t-test for both playback experiment and longitudinal analysis. All analyses were done using SPSS Software for Windows (v. 15 IBM, Chicago, IL, USA,) and graphs were drawn using Sigma plot for Windows (v. 8.02. Systat Software Inc., San Jose, CA, U.S.A.). Data are presented as means ±SD, unless stated otherwise.

## Results

### Playback experiment

All tested birds responded by singing and approaching the speaker. All control birds type matched the song type during the first and/or the second playback trial. Individuals used in total between 1 and 5 different song types during the two playback trials, which represented between 20% and 100% of the previously recorded repertoire. However, there were no differences in the number of different song types used during the playbacks (Own song: 2.2±1.1, Unfamiliar song: 2.7±1.6; *t* = −0.87, *P* = 0.39, *df* = 16). In addition, recordings that were made during the playback tests revealed that none of the birds sang a song type that we had not previously recorded during the first dawn chorus.

**Figure 2:**
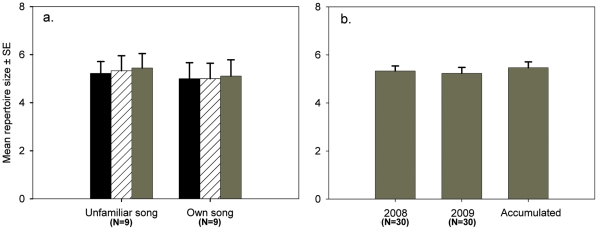
Mean repertoire size ± SE during: a) playback experiment, black bars: first dawn chorus, striped bars: second dawn chorus; grey bars: accumulated repertoire (repertoire size at first dawn chorus + new song types during second dawn chorus); and b) longitudinal analysis in two consecutive years (2008, 2009) and accumulated repertoire (repertoire size 2008 + new song types in 2009).

The results revealed that overall repertoire size was not significantly different between groups (birds that were exposed to their own song and those that were exposed to an unfamiliar song; LMM: *F*_1, 31.02_ = 0.038 *P* = 0.847), phases (first and second dawn chorus; LMM: *F*_1, 30.9_ = 0.01 *P* = 0.921) or their interaction (first dawn chorus: Own = 5±2; Unfamiliar = 5.22±1.48; second dawn chorus: Own = 5±1.936; Unfamiliar = 5.33±1.87; LMM: Group*Phase *F*_1, 30.9_ = 0.01 *P* = 0.921; *N* = 9) (Figure 2a). Age did not have a significant effect (Variance ± SE = 0.3±0.5 *P* = 0.54) and it was excluded from the model. Moreover, individual repertoire size repeatability based on analysis of variance was 0.96 (*P*<0.001).

Birds that heard their own song and birds hearing unfamiliar song did not differ in the number of new or dropped songs (Mann-Whitney U test: New: *Z* = −0.62 *P* = 0.73 Monte Carlo sig: 2-tailed = 1, Dropped: *Z* = 0 *P* = 1, *N* = 9, Monte Carlo sig: 2-tailed = 1). Furthermore, when we compared the first repertoire observed with the accumulated repertoire (First repertoire+new song types) within groups, we did not find any significant difference (Wilcoxon Signed Rank test with exact test: Own: *Z* = −1, *P* = 1 exact sig. 1-tailed = 0.5, *N* = 9; Unfamiliar: *Z* = −1.41, *P* = 0.157 exact sig. 1-tailed = 0.25, *N* = 9) (Figure 2a).

**Figure 3:**
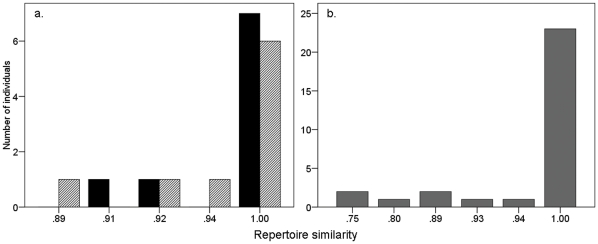
Number of individuals in relation to similarity in repertoire composition between the first and second dawn chorus recorded in a) the playback experiment: black bars: own song group, N = 9; striped bars: unfamiliar song group. N = 9; b) the longitudinal analysis, N = 30.

The analysis of repertoire composition indicated that most of the birds from both the own (7 out of 9) and unfamiliar (6 out of 9) groups sang exactly the same song types after the playback stimulus than before (Figure 3a). Average repertoire similarity between the first and the second dawn chorus was 0.98±0.04 for the own song, and 0.97±0.04 for the unfamiliar song group, and there were no significant differences in the similarity between groups (Unpaired *t*-test: *t* = 0.462 *P* = 0.65).

### Longitudinal analysis

Repertoire size did not differ significantly between years (2008: 5.33±1.15; 2009: 5.23±1.36, *N* = 30; LMM: *F*_1, 46.3_ = 0.12 *P* = 0.731) (Figure 2b). Age did not have a significant effect (Variance ± SE: 0.04±0.07 *P* = 0.623) and was, therefore, excluded from the final model. Additionally, when we compared the repertoire size from 2008 with the accumulated repertoire (2008 + new song types), we did not find any significant difference (Accumulated 5.47±1.36, Wilcoxon Signed Rank test with exact test = −1.63 *P* = 0.1 Monte Carlo sig: 2 tailed = 0.24 *N* = 30) (Figure 2b). Furthermore, individual repertoire size repeatability between years was very high (*r* = 0.91, *P*<0.001).

The analysis of repertoire composition revealed that 23 out of 30 (77%) males in 2009 sang exactly the same song types as in 2008 (Figure 3b) and the average similarity in repertoire composition was 0.96±0.07. We observed that 7 of the 30 recorded males added/dropped songs in 2009, but this change was not related to the presence of new neighbours (change/new neighbour = 6; change/same neighbours = 1; No change/new neighbour = 18; no-change/same neighbours = 5; *χ* ^2^ = 0.186 exact sig 2-sided = 1).

### Relationship between changes in repertoire and repertoire size

Although we observed non-significant changes in repertoire size, we investigated whether those changes were related to the size of the initial repertoire in the males. Males that changed their repertoire during the playback experiment had a tendency for having a larger repertoire during the first dawn chorus recorded (*t*_16_ = 1.78, *P* = 0.094). However, during the longitudinal study, the relationship between change in repertoire and repertoire size was not significant (*t*_8_ = 1.35, *P* = 0.219).

## Discussion

We performed a playback experiment and a longitudinal analysis to replicate previous studies on song repertoire flexibility in the great tit. In contrast to earlier research [17], [18] our results appear to indicate that repertoire size and composition are highly repeatable in the great tit within a breeding season after confrontation with a novel song, and also between years. Our findings, therefore, suggest a very limited repertoire flexibility in our population of the species under study. Only few individuals added/dropped songs from their repertoire, which did not lead to overall changes in repertoire size or composition. The disagreement in results with previous studies can be due to variation in song repertoire learning programmes between the studied populations or by differences in methodological approaches between the studies.

### Population differences

Previous research has shown that populations of the same species may differ in song learning programmes [10]. For instance two different populations of the great reed warbler (*Acrocephalus arundinaceus*) located in Germany and Sweden have been found to differ in learning strategies [39]. Individuals from the Swedish population increased repertoire size with age while this was not the case in the German population. Previous studies on the great tit have also reported differences of song traits at the population level. For instance, the maximum frequency and the average number of notes per song type vary among different populations [40]. Moreover, great tits from nearby forest and city populations seem to differ in frequency and time parameters [41], [42]. This could indicate that repertoire flexibility may be linked to particular populations and conditions. However, because differences in repertoire plasticity and learning programmes among populations have not been studied in detail, the possibility that different populations of the same species may develop a different song learning programme may have been overlooked. As a result, there is no evidence at present that repertoire plasticity is a population-related trait.

On the other hand, population dynamics also seem to play an important role in repertoire composition at population level. Different aspects such as recruitment rate, age structure or number of immigrant birds may have an impact on the song repertoires in the population [15], [17], [38]. However, it still remains unclear whether this could have an impact on the learning programmes.

### Methodological difficulties when studying song repertoire learning in wild populations

Alternatively, another explanation is that differences in methodological approaches among studies may have affected the assessment of repertoire size and composition, and, as such, also the assessment of repertoire plasticity. Unlike Franco & Slabbekoorn [18], we used a colour-ringed nest box breeding population, which enabled us to individually identify the males during all phases of the experiment. As territorial interactions occur every day, birds may move or change singing perches, and territorial take-overs are possible. Consequently, misidentification may occur and may become a confounding factor. In addition, given that previous studies have shown that focal birds respond less aggressively if the simulated intruder during a playback is located outside the territory [43], [44], the playback stimulus in our experiment was placed at a standardized distance from the nest box. Moreover, unlike the two previous studies [17], [18], we tried to employ a standardized recording method taking into account the ‘song ecology’ of great tits. We only recorded complete dawn choruses during egg laying, and the complete playback experiment was performed during this reproductive stage, which is the period of peak singing activity in the great tits [26]. This also enabled us to avoid differences in singing activity due to variation in motivation during the breeding cycle [25], [26], [45], [46].

Our results clearly show that repertoire size and composition are highly repeatable in our population, suggesting on the one hand that great tits may sing their complete repertoire at dawn, and on the other hand, that our recording methods appeared to be highly reliable. The average ± SD repertoire size in our study was 5.33±1.15 in 2008 and 5.23±1.36 in 2009. Although not statistically tested, the observed repertoire size in our population is higher than the repertoire in the population studied by Franco & Slabbekoorn [18] in Leiden (4.25±1.32), and the population studied by McGregor & Krebs [17] in Great Britain (3.07±0.60; [47]). Although we cannot exclude differences at population level, the methodological differences may explain why our repertoire size was larger, and it may also suggest that repertoire size may have been underestimated in the previous studies. This may explain the reported changes in repertoire composition [17] as well as the (surprising) repertoire flexibility [18].

The potential problem of underestimating repertoire size has been recognized previously as a possible confounding factor when studying great tit song. British researchers have already mentioned that they may have spent more time recording individuals with low song production, and that as a consequence, large repertoires may have been underestimated [15], [17], [47]. Moreover, Franco & Slabbekoorn [18] also considered the possibility that they had missed part of the repertoire, since they mentioned that they detected on average 90% of the song types per individual, and when they could only record the minimum number of phrases they were only able to detect 80% of an individual's repertoire. As a consequence, if repertoires are underestimated during the first phase of a study on repertoire plasticity or learning (both longitudinal or experimental in a single year), the ability to recognize ‘new’ songs in the later phases may be confounded by the fact that a part of the repertoire, although present, was not previously recorded.

On the other hand, the time at which recordings were collected, which seems to differ from the one used by McGregor & Krebs [17], may also have played a crucial role in the assessment of repertoire. From the description in their article, it seems that recordings were obtained during daytime singing, while we only recorded individuals during dawn chorus. Differences in singing activity with time of the day or context have been already established for other species. For instance, common nightingales (*Luscinia megarhynchos*) differ in diurnal and nocturnal singing activity [45] and the banded wren (*Thryothorus pleurostictus*) sings longer during dawn chorus and preferentially uses specific song types in territorial interactions [48]. Moreover, some evidence indicates that great tit males only use particular song types in territorial encounters, and males prefer to match the song of the neighbours [22], [49]. If great tits only use particular song types during daytime singing due to the territorial interactions, it would be possible to miss part of the repertoire while making only daytime recordings, which could also lead to an underestimation of the repertoire.

Context dependent use of song repertoires could also have an effect on repertoire assessment during dawn chorus recordings. If great tit males use specific song types depending on the context, there is still a possibility that some song types are not used during dawn chorus. We think however that this is highly unlikely. Firstly, none of the experimental males sang a song type that we had not previously recorded during the first dawn chorus during the playback experiment, which was carried out after the dawn chorus. Secondly, from all the males of which we obtained full dawn chorus recordings, we have never detected ‘new’ song types during daytime recordings (unpublished data).

#### Social interactions and repertoire flexibility during adulthood

Although a limited number of individuals in our population added/dropped song types after confrontation with playbacks or between years, we did not find significant differences between birds that heard their own song or the song of an unfamiliar individual during the playback experiment. Furthermore, the observed changes in the longitudinal study were not related to the presence of new neighbours (as mentioned in McGregor & Krebs [17]). We observed however, that the individuals with changes in their repertoire during the playback experiment tended to have a larger repertoire during the first phase of our playback experiment. If social interactions have an effect on repertoire composition during adulthood, (as previously suggested [17], [18]), great tits should almost permanently change their song types, since confrontations and territorial interactions take place almost daily during the reproductive period in this species. Moreover, it is well-known that song matching occurs very often in great tits [21], [22], [49]. This would lead to an almost continuous increase or turnover of the repertoire. However, our results conclusively show that this clearly was not the case in our population. Great tits in our population do not appear to change repertoire size or composition as adults and social interactions do not seem to play a role in shaping the repertoire during adulthood. On the contrary, our results rather suggest that the observed changes are linked to large repertoires, which also supports the idea that large repertoires are more easily underestimated, even when complete dawn choruses are recorded.

#### Closed-ended versus open-ended learning

Although there is a lack of laboratory experiments to accurately establish the timing of learning in the great tit, this species was initially assumed to be an age-limited learner [15]. However, previous studies seemed to indicate that the great tits may either be open-ended learners, which experience a succession of sensitive periods throughout life, or age-limited learners, which are able to recall songs from memory and use the repertoire in a very flexible way, singing different song types in different periods, years or contexts [17], [18]. Our results show, however, that such repertoire flexibility does not exist, at least in our population. Despite the fact that we did not use controlled laboratory experiments, our findings seem to be more consistent with the idea that great tits probably memorise their songs (sensitive period) early in life, before the first breeding year and that they do not add songs as adults.

Beyond the discussion of being a closed or an open-ended learner, the previous findings on repertoire plasticity in great tits challenged the traditional assessment of song repertoires and the biological relevance of repertoire size as a measurement of male quality. Our study indicates however, that repertoire plasticity, if existing, is very limited, at least in our population. Therefore, repertoire size and composition remain stable after crystallization in the great tit, and the assessment of repertoire size obtained from a complete dawn chorus recording clearly can be considered as a reliable measure of male quality [23], [30], [47]. Moreover, our results suggest that an adequate and standardized method is necessary to correctly establish the song repertoire under field conditions when studying song-learning programmes. To conclude, although we argue that it is likely that the previously observed changes in repertoire size and composition in the great tit can be explained by methodological differences that could have led to underestimation of the repertoires, we can not totally rule out that the discrepancy in results may be due to differences among populations.
